# Neonatal bloodstream infections in a Ghanaian Tertiary Hospital: Are the current antibiotic recommendations adequate?

**DOI:** 10.1186/s12879-016-1913-4

**Published:** 2016-10-24

**Authors:** Appiah-Korang Labi, Noah Obeng-Nkrumah, Stephanie Bjerrum, Christabel Enweronu-Laryea, Mercy Jemima Newman

**Affiliations:** 1 Department of Microbiology, Korle-Bu Teaching Hospital, P.O. Box 77, Accra, Republic of Ghana; 2 Microbiology Department, School of Biomedical and Allied Health Sciences, College of Health Sciences, University of Ghana, P.O. Box 4326, Accra, Republic of Ghana; 3 Department of Infectious Diseases, Institute of Clinical Research, Odense University Hospital, Sdr. Boulevard 29, Odense C, 5000 Odense, Denmark; 4 Department of Child Health, School of Medicine and Dentistry, College of Health Sciences, University of Ghana, P.O. Box 4326, Accra, Republic of Ghana; 5 Department of Medical Microbiology, School of Biomedical and Allied Health Sciences, University of Ghana, P.O. Box 147, Accra, Republic of Ghana

**Keywords:** Neonates, Bloodstream, Infections, Ghana, Antibiotics, Resistance

## Abstract

**Background:**

Diagnosis of bloodstream infections (BSI) in neonates is usually difficult due to minimal symptoms at presentation; thus early empirical therapy guided by local antibiotic susceptibility profile is necessary to improve therapeutic outcomes.

**Methods:**

A review of neonatal blood cultures submitted to the microbiology department of the Korle-Bu Teaching Hospital was conducted from January 2010 through December 2013. We assessed the prevalence of bacteria and fungi involved in BSI and the susceptibility coverage of recommended empiric antibiotics by Ghana Standard Treatment guidelines and the WHO recommendations for managing neonatal sepsis. The national and WHO treatment guidelines recommend either ampicillin plus gentamicin or ampicillin plus cefotaxime for empiric treatment of neonatal BSI. The WHO recommendations also include cloxacillin plus gentamicin. We described the resistance profile over a 28-day neonatal period using multivariable logistic regression analysis with linear or restricted cubic splines.

**Results:**

A total of 8,025 neonatal blood culture reports were reviewed over the four-year period. Total blood culture positivity was 21.9 %. Gram positive organisms accounted for most positive cultures, with coagulase negative staphylococci (CoNS) being the most frequently isolated pathogen in early onset infections (EOS) (59.1 %) and late onset infections (LOS) (52.8 %). Susceptibility coverage of early onset bacterial isolates were 20.7 % to ampicillin plus cefotaxime, 32.2 % to the combination of ampicillin and gentamicin, and 71.7 % to cloxacillin plus gentamicin. For LOS, coverage was 24.6 % to ampicillin plus cefotaxime, 36.2 % to the combination ampicillin and gentamicin and 63.6 % to cloxacillin plus gentamicin. Cloxacillin plus gentamicin remained the most active regimen for EOS and LOS after exclusion of BSI caused by CoNS. For this regimen, the adjusted odds of resistance decreased between 12-34 % per day from birth to day 3 followed by the slowest rate of resistance increase, compared to the other antibiotic regimen, thereafter until day 28. The trend in resistance remained generally unchanged after excluding data from CoNS. Multidrug resistant isolates were significantly (*p*-value <0.001) higher in LOS (62.4 %, *n* = 555/886) than in EOS (37.3 %, *n* = 331/886).

**Conclusions:**

There is low antibiotic susceptibility coverage for organisms causing neonatal bloodstream infections in Korle-Bu Teaching Hospital when the current national and WHO recommended empiric antibiotics were assessed. A continuous surveillance of neonatal BSI is required to guide hospital and national antibiotic treatment guidelines for neonatal sepsis.

**Electronic supplementary material:**

The online version of this article (doi:10.1186/s12879-016-1913-4) contains supplementary material, which is available to authorized users.

## Background

The neonatal age represents a critical period in the life of an infant. The immature state of their immune system predisposes them to infections [1]. Of these, bloodstream infections (BSI) are the most important because of their association with increased cost, morbidity and mortality [1–3]. In Africa, neonatal bloodstream infection rates are estimated to be 20 per 1000 live births’; mainly ascribed to poor intra-partum and postnatal practices [4, 5]. Neonatal bloodstream infections may be of early or late onset. Early onset neonatal BSI (i.e. infections occurring within first 48 h of life) [3] is mostly due to vertical transmission from maternal flora during the perinatal period [6]. Group B Streptococcus is the typical organism isolated during this period in developed countries although other bacteria do play a role [3]. Late onset neonatal BSI (infections occurring past 48 h after birth) [3] are caused by organisms from the hospital or home environment [4, 6]. Commonly associated pathogens are *Staphylococcus* species and Gram negative bacteria mainly *Enterobacteriaceae* [1–3].

In neonates early signs and symptoms of BSI are minimal, making it difficult to diagnose [1, 3]. This means empirical antibiotic management of suspected BSI must be started immediately to reduce associated morbidity and mortality. The antibiotic of choice must cover commonly isolated organisms, and must take into account their known local antibiotic susceptibility patterns. However in developing countries, antimicrobial therapy is usually based on international recommendations without adaptation to local susceptibility patterns; although it has been shown that evidence based treatment improves treatment outcomes [7]. Rising incidence of multi-drug resistant bacteria worldwide [8], and the variability of antibiotic susceptibility patterns by location, means that local antibiotic susceptibility profiles are invaluable to effective empiric antibiotic therapy.

Very few published data on neonatal bloodstream infections exist in Ghana and the West African sub-region. A study conducted among neonates in a major teaching Hospital in Ghana, described decreasing susceptibility of Gram negative organisms to aminoglycosides and cephalosporins [5]. The evolving nature of antibiotic resistance means that there is the need to regularly update antibiotic susceptibility data to guide empiric therapy. The aim of this study was to describe bacterial and fungal isolates from neonatal blood cultures processed at the Microbiology Department of the Korle-Bu Teaching Hospital (KBTH) over a four year period (2010–2013); with emphasis on antibiotic susceptibility patterns, multi-drug resistant isolates and coverage of antibiotics suggested by the current national standard treatment guidelines [9] and the World Health Organisation (WHO) [10] for treating neonatal sepsis. This information may contribute to improved management of neonatal sepsis and the development of local policies based on local epidemiological information.

## Methods

### Study design and setting

We conducted a retrospective review of neonatal blood cultures over a 4-year period from January 2010 through December 2013. Given that the current definition of neonatal BSI incorporate time thresholds that delimit infections between 0 and 28 days after birth, only blood cultures with information on age of patients at time of sampling were considered for review and analyses. Blood culture reports of newborn infants were collated from the bacteriology unit of the Microbiology Department of KBTH, which processes over 40,000 clinical cultures annually. We included blood culture results of neonates primarily receiving healthcare at the KBTH (*n* = 7,594) or secondarily referred to the laboratory from other health facilities for microbiological investigations (*n* = 431). Blood cultures from KBTH were from the Babies Unit and neonatal intensive care units of the Department of Child Health. The Department of Child Health has a 55-bed neonatal intensive care unit; and a 40-bed Babies Unit for neonatal cases referred to KBTH [11]. The hospital annually records about 11,000 live births; and provides referral healthcare services to an estimated population of 24 million Ghanaians [11].

### Laboratory procedures

Over the period under review the laboratory performed 8,025 neonatal blood cultures. For neonatal patients, the guideline is to inject between 1-3 mL of blood directly into Paediatric Bactec® culture vials (Becton Dickinson, USA). These are then submitted for laboratory diagnosis. Cultures are processed with the BACTEC 9240 blood culture system (Becton Dickinson, NJ, USA) according to manufacturer’s instructions. Subcultures are typically made on to blood, chocolate, MacConkey and Saboraund agars; and incubated aerobically at 37 °C for 20 h. Identification of bacterial isolates was performed by Gram-stain microscopy and with routine biochemical methods. Bacteria speciation was done with the BBL Crystal identification system (Becton Dickinson, NJ, USA). Antibiotic susceptibility testing was performed using the Kirby Bauer Disc diffusion method in accordance to Clinical and Laboratory Standards Institute guidelines. Positive blood cultures for yeast and non-yeast fungi were identified on the basis of macromorphology (including estimation of radial growth on selected media) and microscopic examinations of sexual reproduction, conidiogenesis, and hyphal branching. Routinely at the laboratory, positive blood cultures are evaluated to determine whether they represent true bacteraemia, fungaemia, or contamination - they are each reviewed by the Clinical Microbiologist for decisions on the significance of the organism and the appropriate antibiotic susceptibility data to report. The Clinical Microbiologists have access to patients’ clinical information.

### Review and analysis

Study data were collected by physician-assisted review of laboratory records. Information extracted included gender, age and year of infection, the isolated organism and antibiotic susceptibility data. For purposes of this study, positive blood cultures were those with bacteria or fungi (i) recovered and for which antibiotic susceptibility tests were performed (if isolates were bacteria); and (ii) identified as the first isolate per patients within the study period. If more than one organism was isolated from a single culture this was classified as a poly-microbial infection. Certain organisms, including *Micrococcus species*, *Bacillus species*, and diptheroids were classified as contaminants. Coagulase negative *Staphylococci* (CoNS) because of their possible role as pathogens in this age group were included in the overall data for analysis except where specified in the laboratory records as contaminants with no antimicrobial susceptibility data. Neonatal BSI was defined by at least one set of positive blood culture for bacteria or fungi in an infant 28 days old or younger. Bloodstream infections were sub-classified into early onset (EOS) if it occurred <48 h and late onset (LOS) if it occurred 2–28 days after birth. Positive cultures were further categorized into Gram-positive bacteria, Gram-negative organisms, and fungi.

#### Antimicrobial susceptibility

Empirical choice of antibiotics at the KBTH is varied and may involve ampicillin, cloxacillin, gentamicin, amikacin, or ceftotaxime in various combinations. However the national and WHO treatment guidelines recommend either ampicillin plus gentamicin or ampicillin plus cefotaxime for empiric treatment of neonatal BSI [9]. The WHO recommendations also include cloxacillin plus gentamicin [10]. Susceptibility of bacterial isolates to the recommended regimen was evaluated based on reported antibiogram. Isolates were described as susceptible to the combination regimens if they were reported susceptible in vitro to either one or both of the antibiotics recommended. Susceptibility profiles of isolates to different treatment regimens were repeated after excluding BSI with CoNS. We assessed the occurrence of multidrug resistant bacteria between early and late onset infections, focusing on six epidemiological bacterial pathogens: vancomycin resistant *Enterococcocus* species (VRE) [based on in vitro susceptibility to vancomycin disk (30ug)], methicillin resistant *Staphylococcus aureus* (MRSA) [based on in vitro susceptibility to cefoxitin disk (30ug)], penicillin resistant streptococci (PRS) [based on in vitro susceptibility to ampicillin disk (10ug)], cephalosporin resistant enterobacteria (Ceph-R Ent) [based on in vitro susceptibility to cefotaxime disk (30ug)], multi-drug resistant *Pseudomonas* species (MDR Ps.) and multi-drug resistant *Acinetobacter* species (MDR Act). Multidrug resistant (MDR) phenotype was defined according to the international standard definitions for acquired resistance, and relative to the panel of antibiotics tested for each isolate, as in vitro non-susceptibility to ≥1 agent in ≥3 antimicrobial categories: penicillins, cephalosporins, beta-lactamase inhibitor combinations, fluoroquinolones, aminoglycosides, chloramphenicol, folate pathway inhibitors, tetracyclines, macrolides and glycopeptides [12].

### Statistics

Study data was captured into Microsoft Excel, and exported into Statistical Package for Social Sciences (SPSS, Version 20.0) for editing and statistical analyses. Where appropriate, data were compared between four consecutive study periods (2010 through 2013) so that trends could be ascertained. Comparisons between categorical data were conducted with Fisher’s exact test or Chi-square with Marascuilo’s post hoc tests for multiple comparisons. Changes in incidence over time were analyzed by Chi-square test for trend. Continuous data were compared using students’ *t*-test with analyses of variance (ANOVA) for multiple comparisons. A multivariable logistic regression with linear or restricted cubic splines was used to model the association between the proportion of resistant bacteria causing BSI and the age of neonates at the time of blood culture sampling. The number and placement of spline knots was based on a previously published method by Blackburn et al., 2014 [13], using ‘*mkspline* functions’ created in Stata version 12 (StataCorp., USA). Each linear spline incorporated one, two, three or four knots. Restricted cubic splines included four knots. Logistic regression models with the best fit were chosen based on the Akaike’s Information Criterion (AIC). Models with least AIC values were preferred. Organisms, year of infections and patients’ gender were included in the models as cross-factor random effects, continuous and binary variables, respectively. The likelihood ratio test was used to determine the statistical significance for outcomes in the regression models. Point estimates of statistical significance were indicated with 2-tailed *p*-values <0.05. Logistic regression analysis were repeated after excluding BSI with CoNS.

## Results

From January 2010 through December 2013, we reviewed 8025 blood culture reports of neonates. Contaminants accounted for 3.6 % (*n* = 294); and these were more prevalent (*p*-value = 0.001) in newborns <48 h old (17.2 %; *n* = 151/875) than in neonates 2–28 days old (12.1 %; *n* = 143/1182). Blood culture positivity was 21.9 % (*n* = 1763/8025), and remained significantly higher among neonates receiving care at KBTH (22.5 %, *n* = 1712/7594) compared to those referred to the laboratory from elsewhere (11.8 %, *n* = 51/431). In the proceeding analysis however, we have disregarded comparing results between the two sources of blood cultures due to few true positive cultures from the latter.

### Isolates from neonatal BSI

Ranked distributions for reported organisms are presented in Table 1 for EOS and LOS. The data show a predominance (*p*-value <0.001) of LOS (13.0 %, *n* = 1039/8025) over EOS (9.0 %, *n* = 724/8025). Gram-positive organisms accounted for the majority of BSI and were significantly (*p*-value =0.001) more prevalent in EOS (84.2 %, *n* = 610/724) compared to LOS (78.1 %, *n* = 811/1039). Coagulase negative *Staphylococci* were the most frequent organism (EOS: 59.1 % > LOS: 52.8 %; *p*-value = 0.005), followed by *Staphylococcus aureus* (EOS: 10.5 % < LOS: 15.3 %; *p*-value = 0.003) and *Streptococcus* species (EOS: 8.1 % > LOS: 5.6 %; *p*-value = 0.003). For EOS *Citrobacter* (3.2 %, *n* = 23/724) and *Enterobacter* (2.6 %, *n* = 19/724) species were the most frequently reported Gram negative bacteria. *Citrobacter* species accounted for the majority of Gram negative bacteria reported in LOS (4.3 %, *n* = 44/1039). There was a trend towards increased incidence of CoNS in EOS, from 55.3 % in 2010 to 65.4 % in 2013 (Chi- square for trend analysis, *p*-value <0.001). In LOS, we observed a rise in *Candida* species from 0 in 2010 to 0.4 % (*n* = 1/261) in 2011, 0.6 % (*n* = 2/352) in 2012 and 3.3 % (*n* = 5/151) in 2013 (Chi- square for trend, *p*-value <0.001).

**Table 1:** Bacteria and fungi isolates from blood cultures of neonates <48 h and 2–28 days old

Organism	Total		EOS				*X* ^2^ trend *p*-value	LOS				*X* ^2^ trend *p*-value
			Number of isolates (%)					Number of isolates (%)				
	EOS	LOS	2010	2011	2012	2013		2010	2011	2012	2013	
Gram negatives	109 (14.9) ^a^	220 (21.7) ^b^	33 (14.0)	16 (11.1)	45 (17.2)	15 (17.8)	0.219	63 (22.9)	41 (15.7)	80 (22.7)	36 (20.5)	0.541
*Enterobacteriacea*	79 (10.9)	160 (15.3)	28 (11.9)	8 (5.5)	35 (13.4)	8 (9.5)	0.879	45 (16.4)	28 (10.7)	61 (17.3)	26 (17.9)	0.444
*Escherichia coli*	11 (1.5)	14 (1.3)	2 (0.9)	1 (0.7)	6 (2.2)	2 (2.4)	0.183	5 (1.8)	5 (1.9)	10 (2.8)	4 (2.6)	0.450
*Klebsiella* species	10 (1.4)	21 (2.0)	3 (1.2)	1 (0.7)	5 (1.9)	1 (1.2)	0.801	5 (1.8)	1 (0.4)	11 (3.1)	4 (2.6)	0.210
*Kluyvera* species	7 (0.9)	6 (0.6)	5 (2.1)	0	1 (0.4)	1 (1.2)	0.109	1 (0.3)	1 (0.4)	1 (0.2)	3 (2.0)	0.187
*Citrobacter* species	23 (3.2)	44 (4.3)	7 (2.9)	3 (2.1)	11 (4.2)	2 (2.4)	0.785	16 (5.8)	10 (3.8)	12 (3.4)	6 (4.0)	0.199
*Enterobacter* species	19 (2.6)	40 (3.9)	6 (2.5)	2 (1.4)	10 (3.8)	1 (1.2)	0.927	14 (5.1)	4 (1.5)	19 (5.4)	3 (2.0)	0.520
*Proteus* species	5 (0.7)	7 (0.6)	3 (1.2)	1 (0.7)	1 (0.4)	0	0.097	0	2 (0.6)	3 (0.9)	2 (1.3)	0.144
*Salmonella* species	3 (0.4)	10 (0.9)	1 (0.4)	0	1 (0.4)	1 (1.2)	0.698	3 (1.1)	3 (1.1)	3 (0.9)	1 (0.7)	0.506
*Serratia* species	1 (0.4)	8 (0.7)	1 (0.4)	0	0	0	0.089	1 (0.3)	2 (0.8)	2 (0.6)	3 (2.0)	0.216
*Acinetobacter* species	16 (2.2)	43 (4.1)	3 (1.2)	4 (2.7)	4 (1.5)	5 (5.9)	0.131	15 (5.4)	7 (2.6)	13 (3.7)	8 (5.3)	0.740
*Pseudomonas* species	11 (1.5)	16 (1.5)	2 (0.8)	2 (1.3)	5 (1.9)	2 (2.4)	0.300	3 (1.1)	6 (2.3)	5 (1.4)	2 (1.3)	0.934
Others	3 (0.4)	1 (0.09)	0	2 (1.3)	1 (0.4)	0	0.865	0	0	1	0	0.895
Gram positives	610 (84.2) ^b^	811 (78.0) ^a^	200 (85.1)	127 (88.2)	214 (81.9)	69 (82.1)	0.241	212 (77.1)	219 (83.9)	270 (76.7)	114 (75.5)	0.386
*Enterococcus* species	43 (5.9)	36 (3.4)	32 (13.7)	3 (2.1)	6 (2.3)	2 (2.4)	<0.001	23 (8.4)	5 (1.9)	6 (1.7)	2 (1.3)	<0.001
*Micrococcus* species	1 (0.4)	5 (0.4)	1 (0.4)	0	0	0	0.089	0	1 (0.4)	2 (0.6)	2 (1.3)	0.108
*Staphylococcus aureus*	76 (10.5)	159 (15.3)	24 (10.2)	20 (13.9)	24 (9.2)	8 (9.5)	0.570	43 (15.6)	51 (19.5)	46 (13)	19 (12.6)	0.142
CoNS	431 (59.5)	549 (52.8)	130 (55.3)	94 (65.2)	152 (58.2)	55 (65.4)	<0.001	133 (48.4)	150 (57.5)	195 (55.3)	71 (47.0)	0.844
*Streptococcus species*	59 (8.1)	58 (5.6)	13 (55.3)	10	32 (12.3)	4 (4.8)	0.162	13 (4.7)	12 (4.6)	21 (6.0)	4 (2.6)	0.698
*Streptococcus pneumoniae*	3 (0.4)	9 (0.9)	2 (0.8)	0	1 (0.4)	0	0.199	2 (0.6)	1 (0.4)	3 (0.9)	3 (2.0)	0.988
*Streptococcus pyogenes*	3 (0.4)	9 (0.9)	2 (0.8)	0	1 (0.4)	0	0.199	2 (0.6)	1 (0.4)	3 (0.9)	3 (2.0)	0.295
*Streptococcus agalactiae*	5 (0.7)	7 (0.6)	2 (0.8)	1 (0.7)	1 (0.4)	1 (1.2)	0.715	1 (0.3)	2 (0.8)	3 (0.9)	1 (1.3)	0.726
Viridans Streptococci	22 (3.0)	16 (1.5)	2 (0.8)	5 (3.5)	13 (4.9)	2 (2.4)	0.073	2 (0.6)	3 (1.1)	7 (1.9)	4 (2.6)	0.102
Other Streptococci	26 (3.6)	17 (1.6)	5 (2.1)	4 (2.8)	16 (6.1)	1 (1.2)	0.288	6 (2.0)	4 (1.5)	5 (1.4)	2 (1.3)	0.378
*Streptomyces* species	0	4 (0.3)	0	0	0	0	-	0	1 (0.4)	0	3 (2.0)	0.049
Fungi	5 (0.7) ^a^	8 (0.7) ^a^	5 (2.1)	1 (0.7)	2 (0.8)	0	0.053	0	1 (0.4)	2 (0.6)	6 (3.9)	<0.001
*Aspergillus* species	2 (0.2)	0	1 (0.4)	0	1 (0.4)	0	0.480	0	0	0	0	-
*Candida albicans*	3 (0.4)	8 (0.7)	1 (0.4)	1 (0.06)	1 (0.4)	0	0.467	0	1 (0.4)	2 (0.6)	5 (3.3)	<0.001
Total	724 (41.1)	1039 (58.9)	235	144	261	84		275	261	352	151	

*Differing superscripts across columns indicate significant difference at *p*-value <0.05; EOS, early onset infections; LOS, late onset infections; CoNS, coagulase negative staphylococci. Other Gram negatives included *Neisseria* species, *Stenophromonas* species and *Moraxella* species; Other Streptococci included *S. mitis, S. salivarius, S. oralis, S sanguinis group, S bovis, S milleri, S. anginosus, S. constellatus and S. intermedius*

### Antibiotic susceptibility

Table 2 compares in vitro susceptibility of bacterial isolates to empiric antimicrobials used for treatment of neonatal sepsis. In EOS, the susceptibility of all isolates was 20.7 % to ampicillin plus cefotaxime, 32.2 % to the combination of ampicillin and gentamicin and 71.6 % to cloxacillin plus gentamicin (Chi-square for trend, *p*-value <0.001). In Table 3 the antimicrobial profiles for all bacteria isolated from LOS showed that susceptibility was 24.6 % to ampicillin plus cefotaxime, 36.2 % to the combination ampicillin and gentamicin and 63.6 % to cloxacillin plus gentamicin (Chi-square for trend, *p*-value <0.001). For each treatment regimen, there was no significant difference in proportion of susceptible isolates between EOS and LOS. Overall, the combination of cloxacillin and gentamicin remained the most active regimen for EOS and LOS even after exclusion of BSI by CoNS. In EOS, when the analysis excluded data on CoNS, the susceptibility coverage of cloxacillin/gentamicin decreased by 7.7 % but this did not achieve statistical significance (*p*-value = 0.081). In LOS (Table 3), susceptibility to cloxacillin plus gentamicin significantly (*p*-value = 0.011) reduced from 63.6 % (*n* = 570/896) to 55.8 % (*n* = 204/365) after the exclusion of CoNS.

**Table 2:** Susceptibility (of bacteria from blood cultures taken from neonates less than 48 h old) to antimicrobials used for empiric treatment of neonatal BSI

Organisms	Susceptibility							
		Ampicillin + Gentamicin (A)		Ampicillin + Cefotaxime (B)		Cloxacillin + Gentamicin (C)		Regimen coverage
	Number of isolates	Number Tested (%)	Number susceptible (%)	Number Tested (%)	Number susceptible (%)	Number Tested (%)	Number susceptible (%)	
Gram negatives	109	101 (92.6)	38 (37.6)^a^	68 (62.4)	32 (47.1)^a^	75 (68.8)	52 (69.3)^b^	C > B = A; *p* = 0.001
*Enterobacteriacea*	79	74 (93.7)	24 (32.4)	55 (69.6)	27 (49.1)	53 (67.1)	37 (69.8)	
*Acinetobacter* species	16	16 (100)	10 (62.5)	13 (81.2)	5 (38.4)	13 (81.2)	8 (61.5)	
*Pseudomonas* species	11	11 (100)	4 (36.4)	-	-	9 (81.8)	7 (77.7)	
Others	3							
Gram positives	610	511 (83.8)	159 (31.1)^b^	356 (58.4)	56 (15.7)^a^	211 (34.6)	153 (72.5)^b^	C > A > B; *p* = 0.001
*Enterococcus* species	43	37 (86.1)	8 (21.6)	37 (86.1)	7 (18.9)	-	-	
*Staphylococcus aureus*	76	67 (88.2)	34 (50.7)	59 (77.6)	5 (8.5)	69 (90.7)	46 (66.7)	
CoNS	431	376 (87.2)	107 (28.5)	226 (52.4)	26 (11.5)	111 (25.7)	93 (83.7)	C > A > B; *p* = 0.001
*Streptococcus* species	59	31 (52.5)	10 (32.3)	34 (57.6)	18 (30.5)	31 (52.5)	14 (45.2)	
Others	1							
Total	719	612 (85.1)	_a_197 (32.2)^b^	424 (58.9)	_a_ 88 (20.7) ^a^	286 (40.0)	_a_205 (71.7)^c^	C > A > B; *p* = 0.001
Total excluding CoNS	288	236 (81.9)	_a_90 (38.1)^a^	198 (68.8)	_b_ 62 (31.3)^a^	175 (60.7)	_a_112 (64.0)^b^	C > B = A; *p* = 0.001
Polymicrobial infections	20	19 (95.0)	4 (21)^a^	18 (90.0)	2 (11.1)^a^	20 (100.0)	5 (25.0)^a^	C = B = A; *p* = 0.540
2 organisms	17	16 (94.1)	1 (6.3)	15 (88.2)	1 (6.7)	17 (100)	4 (23.5)	
3 organisms	3	3 (100)	3 (100)	3 (100)	1 (33.3)	3 (100)	1 (33.3)	

Based on in-vitro susceptibility. Data on isolates with insufficient numbers (*n* ≤ 5) not shown. For polymicrobial infections, all isolates were susceptible to at least one of the treatment antibiotics

A, Ampicillin plus Gentamicin; B, Ampicillin plus cefotaxime; C, Cloxacillin plus gentamicin; CoNS, Coagulase negative *Staphylococcus* species. Other Gram-negatives included *Neisseria* species (*n* = 1), *Stenophromonas* species (*n* = 1) and *Moraxella* species (*n* = 1). Other Gram-positives included *Micrococcus* species

Differing superscripts across row denotes differences in the proportion of susceptible isolates to antimicrobial regimen. Differences in proportions computed by Chi square with Marascuilo’s Post Hoc Multiple proportion comparisons at *p*-value <0.05. Differing subscripts within a column denotes differences in the proportion of susceptible isolates to antimicrobial regimen

**Table 3:** Susceptibility (of bacteria from blood cultures taken from neonates between 2 to 28 days) to antimicrobials used for empiric treatment of neonatal septicaemia

Organisms	Number of isolates	Susceptibility						
		Ampicillin + Gentamicin (A)		Ampicillin + Cefotaxime (B)		Cloxacillin + Gentamicin (C)		Regimen coverage
		Number Tested (%)	Number susceptible (%)	Number Tested (%)	Number susceptible (%)	Number Tested (%)	Number susceptible (%)	
Gram negatives	220	200 (90.9)	72 (36.0)^a^	188 (85.4)	92 (48.9)^a^	173 (0.78)	102 (46.3)^b^	C > B = A; *p* = 0.001
*Enterobacteriacea*	160	145 (90.6)	44 (30.3)	149 (93.1)	75 (50.3)	131 (81.8)	78 (59.5)	
*Acinetobacter* species	43	40 (93.0)	22 (55.0)	39 (90.7)	17 (43.6)	31 (72.1)	17 (54.8)	
*Pseudomonas* species	16	15 (93.7)	6 (40.0)	-	-	11 (68.7)	7 (63.6)	
Others	1							
Gram positives	811	762 (93.9)	276 (36.3)^b^	770 (94.9)	144 (18.7)^a^	723 (89.1)	468 (64.7)^c^	C > B < A; *p* = 0.001
*Enterococcus* species	36	29 (80.5)	8 (27.5)	34 (94.4)	9 (26.4)	-	-	
*Staphylococcus aureus*	159	143 (89.9)	64 (44.7)	151 (94.9)	22 (14.6)	142 (89.3)	82 (57.7)	
CoNS	549	539 (98.2)	183 (34.0)^b^	540 (98.3)	97 (17.9)^a^	531 (96.7)	366 (68.9)^c^	C > B < A; *p* = 0.001
*Streptococcus* species	58	51 (97.9)	21 (41.0)	45 (77.5)	16 (35.5)	50 (86.2)	20 (40.0)	
Others	9							
*Total*	1031	962 (93.3)	_a_348 (36.2)^b^	958 (92.9)	_a_236 (24.6)^a^	896 (86.9)	_a_570 (63.6)^c^	C > B < A; *p* = 0.001
Total excluding CoNS	482	423 (87.8)	_a_165 (39.0)^b^	418 (86.7)	_b_139 (33.2)^a^	365 (75.7)	_b_204 (55.8)^c^	C > B < A; *p* = 0.001
Polymicrobial infections	25	16 (64.0)	3 (18.7)	14 (56.0)	3 (21.4)	16 (64.0)	6 (37.7)	C = B = A; *p =* 0.431
2 organisms	22	14 (63.6)	2 (14.2)	12 (54.5)	2 (16.7)	15 (68.2)	5 (33.3)	
3 organisms	3	2 (66.7)	1 (50.0)	2 (66.7)	1 (50.0)	1 (33.3)	1 (100.0)	

Based on in-vitro susceptibility. Data on isolates with insufficient numbers (n ≤ 5) not shown. For polymicrobial infections, all isolates were susceptible to at least one of the treatment antibiotics

A, Ampicillin plus Gentamicin; B, Ampicillin plus cefotaxime; C, Cloxacillin plus gentamicin; CoNS, Coagulase negative *Staphylococcus* species. Other Gram-negatives included *Neisseria* species (n = 1), *Stenophromonas* species (n = 1) and *Moraxella* species (n = 1). Other Gram-positives included *Micrococcus* species (n = 5), and *Sterptomyces* species (n = 4)

Differing superscripts across row denotes differences in the proportion of susceptible isolates to antimicrobial regimen. Differences in proportions computed by Chi square with Marascuilo’s Post Hoc Multiple proportion comparisons at *p*-value <0.05. Differing subscripts within a column denotes differences in the proportion of susceptible isolates to antimicrobial regimen

### Changes in antibiotic susceptibility over 4-year study period

**Fig. 1:**
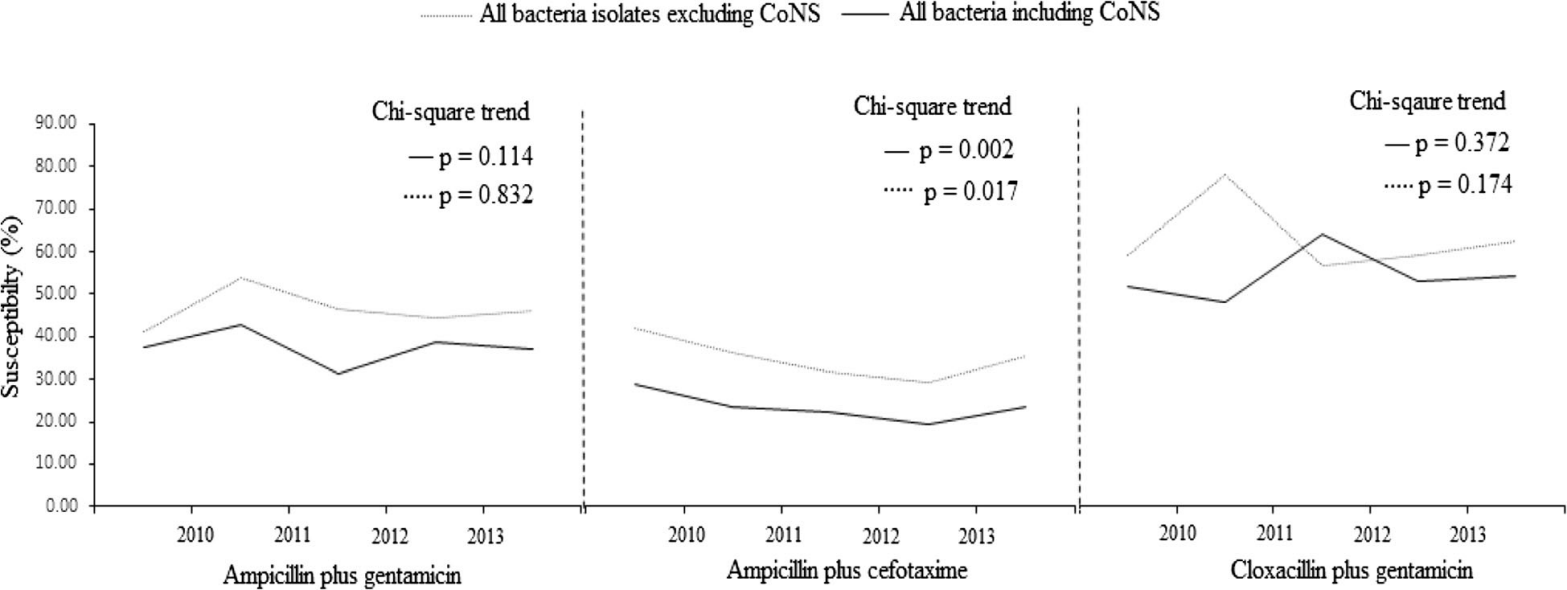
Antibiotic resistance across study years. *CoNS*, *coagulase negative staphylococci*; *%, percentage*. Resistance of blood culture isolates (with or without CoNS) to ampicillin/cefotaxime significantly increased over study period. Resistance to ampicillin/gentamicin and cloxacillin/gentamicin remained relatively unchanged from 2010 through 2013

Resistance of blood culture isolates to ampicillin/gentamicin and to cloxacillin/gentamicin remained relatively unchanged from 2010 through 2013 (Fig. 1). In contrast, we identified significant increase in resistance to ampicillin/cefotaxime during the same study period. The yearly trend in resistance to each regimen remained unchanged after excluding data on CoNS.

### Changes in antibiotic susceptibility over 28-day neonatal period

**Fig. 2:**
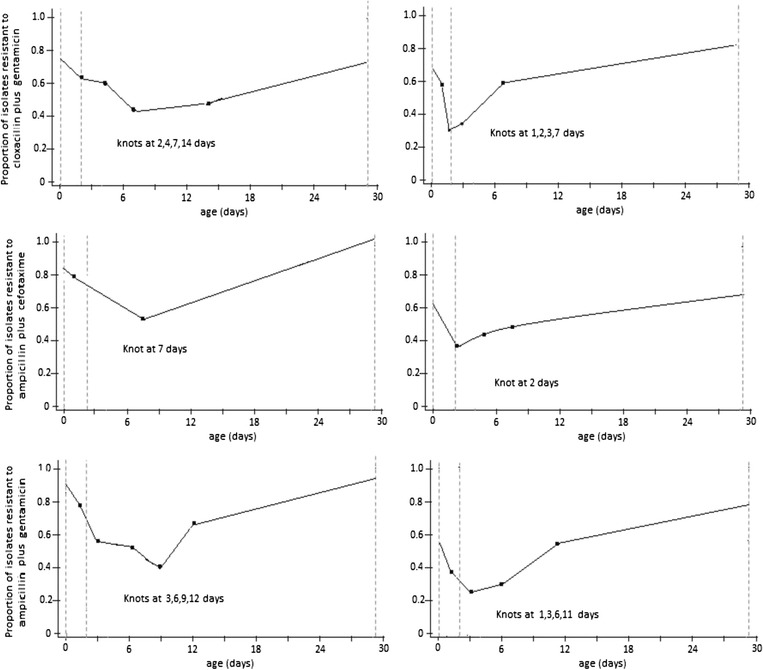
Change in the odds of antibiotic resistance in (i) all bacteria (i) and excluding coagulae negative *Staphylococcus* species (ii) Models are linear spline function estimates shown on the logit scale based on the least Akaike’s Information Criterion (Additional file 1: Table S1)

The relationship between resistance to each empiric antibiotic combinations and neonates’ age was best described by linear spline functions incorporating one or four age breakpoints (knots) (Additional file 1: Table S1). After adjusting for gender, year of infection and bacterial isolates, the odds ratios from multivariable logistic regression showed evidence of association between resistance and age for each antibiotic regimen (*p*-value <0.008 for all antibiotic combinations) (Additional file 1: Table S2). The adjusted odds ratios (AOR) can be interpreted as resistance decreasing at varying rates between 12–26 % per day from birth (day 0) to day 7 for cloxacillin/gentamicin and ampicillin/cefotaxime, and to day 9 for ampicillin/gentamicin — followed by increasing resistance levels thereafter until day 28 (Fig. 2). Ampicillin/gentamicin recorded the slowest rate in decline of antibiotic resistance. The odds of resistance fell by 22 % between birth and day 3 followed by a similar decrease of 22 % per day from days 6–9. The rate of increase in resistance was slowest for cloxacillin/gentamicin — the rate increases each day by 24 % from days 7 through 14 and by 61 % thereafter until 28 days (Fig. 2). The trends in resistance over 28-day period remained generally unchanged after excluding data from CoNS, although increase in adjusted odds of resistance occurred within fewer days after birth (Additional file 1: Table S2). Resistance decreased between 29-49 % per day from birth to day 3 for all antibiotic combinations. The decrease in resistance was greatest for cloxacillin/gentamicin. For this regimen, the odds of resistance fell by 38 % from birth to day 1, with a further 11 % decrease in day 2 followed by increasing resistance thereafter until day 28 (Fig. 2)

### Multidrug resistance

**Fig. 3:**
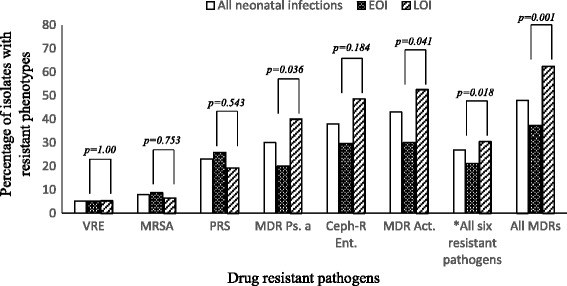
Antimicrobial resistance phenotypes in early and late onset neonatal bloodstream infections. VRE, vancomycin resistant *Enterococci*; MRSA, methicillin resistant *Staphylococcus aureus*; PRS, penicillin resistant Streptococci; Cef-R Ent, cephalosporin resistant *Enterobacteriaceae*; MDR Ps, multidrug resistant *Pseudomonas aeruginosa*; MDR Act, multidrug resistant Acinetobacter species; * All six resistant pathogens is the pooled resistance for all six selected antibiotic drug resistance phenotypes

Figure 3 compares the occurrence of epidemiologically important bacterial phenotypes across EOS and LOS. No carbapenem resistant *Enterobacteriaceae* was isolated. The incidence of Ceph-R Ent. and MDR Act. were significantly higher among LOS than in EOS. Meanwhile, VRE, MRSA, PRS and MDR Ps. were equally prevalent in LOS and EOS. Overall 53 % (*n* = 917/1763) of the total isolates exhibited MDR, accounting for about 50 % (*n* = 886/1763) of total bloodstream infections. Gram negative bacteria had more MDRs (71.7 %; *n* = 236/329) than GPB (47.9 %; *n* = 681/1421) (*p*-value =0.001). The MDRs were less prevalent (*p*-value <0.001) in EOS (37.3 %, *n* = 331/886) compared to LOS (62.4 %, *n* = 555/886). The yearly incidence of all MDRs remained relatively stable from 2010 through 2014 for EOS (Chi-square for trend, *p*-value = 0.284) and LOS (Chi-square for linear trend, *p*-value = 0.531).

## Discussion

The rise in antibiotic resistance worldwide means that empirical therapy guided by local susceptibility is critical for improved therapeutic outcomes. In this study about a quarter of all neonatal blood cultures in our institution over a four year period were positive. We found a high prevalence of Gram-positive organisms which contrasts with previous findings in our hospital [5] and elsewhere [14, 15] where Gram-negative bacteria were the predominant organisms isolated. Coagulase negative *Staphylococcus* was the predominant organism identified in our study. The role played by CoNS in neonatal bloodstream infections is however not clear, as isolation from a single blood culture may represent true bacteraemia or contamination [16, 17]. Coagulase negative *Staphylococcus* have been found to play a significant role in bloodstream infections in neonates with low birth weight and gestational age [16–18] as well as in those with intravascular catheters insitu. In this study however, we were unable to ascertain the percentage of neonates with low birth weight; although anecdotally there is very low use of central catheters among neonates at the KBTH. Others have also suggested that a majority of blood cultures in neonates are performed in the first few days of life because of perceived risk factors of sepsis and not for clinical features of sepsis; and this may account for high rates of contamination with CoNS [19]. Thus it is possible that the high rate of isolation of CoNS from our study may point to high contamination rates from skin flora due to poor skin disinfection techniques. Despite this, other studies have reported high prevalence of CoNS among neonatal blood cultures [1, 2, 20].

Members of the *Enterobacteriaceae* were the commonest Gram negative organisms, with *Citrobacter* species, *Enterobacter* species and *Klebsiella* species accounting for the majority of organisms. This is in accordance with findings from other developing countries [1, 2, 14, 15, 21] as well as previous reports among neonates in KBTH [5]. The major role played by *Enterobacteriaceae* in neonatal sepsis may reflect a high rate of healthcare associated infections among our neonatal population, with these infections likely to have been transmitted from the environment by healthcare personnel and parents [4].

This study confirmed findings of low prevalence of *Streptococcus agalactaie* as a cause of EOS [5] This is contrary to findings in developed countries where EOS is mostly associated with *Streptococcus agalactiae* [3, 6]. However, similar findings of low prevalence have been made in other developing countries [2, 14, 15, 20, 21]. The prevalence of *Streptococcus agalactiae* in this study was low (0.68 %), despite a previous study showing *Streptococcus agalactiae* colonization rate of 19 % among pregnant women [22]. This finding may point to a minimal role played by *Streptococcus agalactiae* in neonatal sepsis in our environment.

The overall susceptibility of blood culture isolates (with and without CoNS) to the antibiotics commonly used showed low susceptibility levels for the recommended regimens of ampicillin/gentamicin and ampicillin/cefotaxime [9, 10]. Cloxacillin/gentamicin had improved coverage over the national treatment guidelines, and remained the most active antibiotic combination despite a moderate coverage of <65.6 % for all bacterial isolates (with or without CoNS). This observation is in agreement with previous studies in Ghana and at Korle-Bu teaching Hospital, that found high resistance to ampicillin, gentamicin and cefotaxime among Gram negative isolates [23–25]. It is also the experience in KBTH that resistance to many routinely used antibiotics is high; and the prevalence is rising [25]. The pattern of resistance further mirrors findings from other developing countries were ampicillin and gentamicin have been found not to be very effective [4]. This suggests that current national antibiotic guidelines for neonatal sepsis may not be appropriate for management of neonatal infections in our study setting. Our results identified a pattern of resistance across neonatal age for all antibiotic combinations. When data excluded CoNS, we observed a decline in resistance for bacteria causing BSI in neonates aged <3 days for each of the antibiotic combinations. The rate in decrease of resistance was highest for cloxacillin/gentamicin. When data included CoNS, our results identified an age (9 days) beyond which antibiotic resistance appear to increase. This rate of increase in resistance was slowest for cloxacillin/gentamicin. Early onset resistance may point to the role played by resistant organisms of maternal origin, whilst increasing resistance over time may be indicative of increased role played by healthcare related infections which is associated with increased chances of antibiotic resistance [3]. This observation is reflected in our finding that GNB (which were more often MDRs; and are frequently associated with hospital flora) were dominant in LOS.

Also the study showed high levels of cefotaxime resistant *Enterobacteriaceae* which is a possible marker for extended spectrum beta lactamase (ESBL) production. However, data on ESBLs were not available for the study isolates. There was also high level of multi-drug resistant *Acinetobacter* species, but low levels of VRE and MRSA, and absence of carbapenem resistant *Enterobacteriaceae*. The KBTH has a higher prevalence (>40 %) of infections caused by ESBL-producers [25, 26] compared to MRSA (<10 %) [27]. High rates of ESBL-producers have been reported in community-acquired and neonatal bloodstream infections elsewhere in Ghana [28]. It is also anecdotal experience that CRE are encountered more often than VRE, but there are no published reports to document this evidence. This study shows that continuous surveillance of bloodstream infections among the neonatal populations in Ghana is needed to inform management strategies and policy which will contribute to the reduction of neonatal mortality from sepsis. The study had some limitations. Our study was retrospective thus affected by missing data including diagnosis of patients at time of sampling. Also, it was not possible to compare in vitro susceptibility testing of recommended antibiotic combinations with in vivo clinical efficacy.

## Conclusion

From our study we observed a blood culture positivity rate of 21.9 % among neonates with most infections being of late onset. Coagulase negative *Staphylococcus* species were the commonly isolated organism in both early and late onset bacteraemia although their exact pathogenic role could not be determined. Whilst members of the *Enterobacteriaceae* (*Citrobacter* species, *Enterobacter* species, *Klebsiella* species) represented the commonest Gram negative organisms isolated. There was very high resistance to ampicillin/gentamicin and ampicillin/cefotaxime combinations among the isolated organism whilst relatively high susceptibility was observed to cloxacillin/gentamicin combination. Continuous surveillance of neonatal bloodstream infections is required to guide hospital and national antibiotic treatment guidelines to improve morbidity and mortality associated with neonatal bloodstream infections.

## Additional file

Additional file 1: Table S1. Logistic regression models using linear or restricted cubic spline function (with the lowest AIC) of neonates’ age. **Table S2.** Changes in the odds of antibiotic resistance with age of neonates to various antibiotic combinations using multivariable logistic regression models with linear spline function. (DOCX 19 kb)

## Abbreviations

μg: Microgram
AKL: Appiah-Korang Labi
ANOVA: Analysis of variance
BSI: Blood stream infections
CEL: Christabel Enweronu-Laryea
Ceph-R Ent: Cephalosporin resistant enterobacteria
CI: Confidence interval
CLSI: Clinical and Laboratory Standards Institute
GNB: Gram-negative bacteria
GPB: Gram-positive bacteria
KBTH: Korle-Bu Teaching Hospital
MDR Act: Multi-drug resistant *Acinetobacter* species
MDR Ps: Multi-drug resistant *Pseudomonas* species
MDR: Multidrug resistance
MDR: Multidrug resistant
MJN: Mercy Jemima Newman
MRSA: Methicillin resistant *Staphylococcus aureus*
NON: Noah Obeng-Nkrumah
PRS: Penicillin resistant streptococci
SB: Stephanie Bjerrum
SPSS: Statistical Package for Social Sciences
VRE: *Enterococcocus* species
VRE: *Enterococcocus* species
