# Supplementary material for: Neonatal bloodstream infections in a Ghanaian Tertiary Hospital: Are the current antibiotic recommendations adequate?
Source: BMC Infect Dis. 2016 Oct 24;16:598. doi: 10.1186/s12879-016-1913-4 (PMC5078915; doi:10.1186/s12879-016-1913-4)
Supplement: Additional file 1: Table S1. — Logistic regression models using linear or restricted cubic spline function (with the lowest AIC) of neonates’ age. Table S2. Changes in the odds of antibiotic resistance with age of neonates to various antibiotic combinations using multivariable logistic regression models with linear spline function. (DOCX 19 kb) [file 12879_2016_1913_MOESM1_ESM.docx]

Supplementary Table 1. Logistic regression models using linear or restricted cubic spline function (with the lowest AIC) of neonates’ age

| Logistic models | Number of Knots | Amp/Gen | |  | Amp/Cef | |  | Clo/Gen | |  |
| --- | --- | --- | --- | --- | --- | --- | --- | --- | --- | --- |
|  |  | Knot days | AIC |  | Knot days | AIC |  | Knot days | AIC |  |
| All bacteria isolates | |  |  |  |  |  |  |  |  |  |
| Linear | 1 | 7 | 896.45 |  | 7 | 869.13 |  | 5 | 833.61 |  |
|  | 2 | 3,9 | 895.61 |  | 4, 7 | 873.22 |  | 1, 6 | 832.63 |  |
|  | 3 | 1,2, 10 | 894.11 |  | 2, 5, 7 | 875.24 |  | 1, 3, 7 | 828.77 |  |
|  | 4 | 3,6,9,12 | 892.82 |  | 2, 4, 7, 14 | 876.36 |  | 2,4,7,14 | 826.51 |  |
| Cubic | 4 | 2,4,9,12 | 898.76 |  | 2,3, 8, 10 | 878.98 |  | 1,4,9,12 | 835.82 |  |
|  |  |  |  |  |  |  |  |  |  |  |
| All bacteria isolates excluding  CoNS | | | | | | |  |  |  |  |
| Linear | 1 | 3 | 582.22 |  | 3 | 571.33 |  | 2 | 593.22 |  |
|  | 2 | 2,7 | 582.72 |  | 1,6 | 571.69 |  | 2,5 | 591.45 |  |
|  | 3 | 1,3,5,7 | 583.66 |  | 1,4,7 | 572.43 |  | 1,3,5,11 | 591.12 |  |
|  | 4 | 1,3,6,11 | 584.92 |  | 1,4,9,12 | 573.51 |  | 1,2,3,7 | 590.53 |  |
| Cubic | 4 | 2,3,8,11 | 585.83 |  | 1,3,6,11 | 578.04 |  | 1,3,6,10 | 595.84 |  |
|  |  |  |  |  |  |  |  |  |  |  |

AIC, Akaike’s Information Criterion.

Black fonts in dark background show knot combinations with the least AIC for various knot combinations used to assess whether the association between neonatal age and bacterial resistance was confounded by other factors (gender, year of infection, infecting organism).

Amp, Ampicillin; Gen, Gentamicin; Cef, cefotaxime; Clo, cloxacillin; Gen, Gentamicin.

CoNS, Coagulase negative *Staphylococcus* species

Supplementary Table 2. Changes in the odds of antibiotic resistance with age of neonates to various antibiotic combinations using multivariable logistic regression models with linear spline function

| Antibiotic regimen | All bacteria isolates | | | | |  | Bacteria excluding CoNS | | | | |
| --- | --- | --- | --- | --- | --- | --- | --- | --- | --- | --- | --- |
|  | Logistic model | Age in days | AOR | 95%CI | *LRT*  *p*-value |  | Logistic model | Age in days | AOR | 95%CI | *LRT*  *p*-value |
|  |  |  |  |  |  |  |  |  |  |  |  |
| Ampicillin/ gentamicin | Linear splines at days 3,6,9,12 | 0-3 | 0.78 | 0.66-0.88 | <0.001 |  | Linear spline with knot at days 1,3,6,11 | 0-1 | 0.64 | 0.43-0.81 | <0.001 |
|  |  | 3-6 | 0.80 | 0.68-0.91 |  |  |  | 1-3 | 0.69 | 0.48-0.79 |  |
|  |  | 6-9 | 0.78 | 0.65-0.89 |  |  |  | 3-6 | 1.58 | 1.31-1.72 |  |
|  |  | 9-12 | 2.13 | 1.87-2.68 |  |  |  | 6-11 | 1.84 | 1.72-1.91 |  |
|  |  | 12-28 | 1.61 | 1.54-1.79 |  |  |  | 11-28 | 1.56 | 1.37-1.71 |  |
| Ampicillin/ cefotaxime | Linear spline with knot at day 7 | 0-7 | 0.74 | 0.58-0.87 | 0.008 |  | Linear spline with knot at day 2 | 0-3 | 0.67 | 0.51-0.78 | 0.005 |
|  |  | 7-28 | 1.71 | 1.62-2.01 |  |  |  | 2-28 | 1.26 | 1.17-1.42 |  |
|  |  |  |  |  |  |  |  |  |  |  |  |
|  |  |  |  |  |  |  |  |  |  |  |  |
|  |  |  |  |  |  |  |  |  |  |  |  |
| Cloxacillin/ gentamicin | Linear splines with knot at days 2,4,7,14 | 0-2 | 0.76 | 0.56-0.93 | 0.002 |  | Linear splines with knot at days 1,2,3,7 | 0-1 | 0.62 | 0.51-0.82 | 0.005 |
|  |  | 2-4 | 0.88 | 0.72-0.98 |  |  |  | 1-2 | 0.51 | 0.34-0.77 |  |
|  |  | 4-7 | 0.74 | 0.64-0.88 |  |  |  | 2-3 | 1.35 | 1.16-1.49 |  |
|  |  | 7-14 | 1.24 | 1.09-1.31 |  |  |  | 3-7 | 2.25 | 2.11-2.42 |  |
|  |  | 14-28 | 1.61 | 1.53-1.79 |  |  |  | 7-28 | 1.28 | 1.17-1.39 |  |

AOR, Adjusted odds ratio; CI, confidence interval; Num.,number; obs. observations; *p*-value calculated as likelihood ratio test for age function.

Multivariable logistic regression adjusted for gender, year of infection and infecting organism.

CoNS, Coagulase negative *Staphylococcus* species
